# Editorial

**Published:** 1842-09-17

**Authors:** 


THE MEDICAL EXAMINER.

PHILADELPHIA, SEPTEMBER 17, 1842.

### MISCELLANEOUS NOTICES.

*Medical Department of Pennsylvania College.*—We have received the third annual annuncement of this institution, which has now been in existence three years, and completed three sessions or annual courses of lectures.

Within this period, it appears that it has numbered upwards of two hundred and fifty students, and conferred the degree of M. D., upon ninety-eight graduates.

*Medical College of Ohio.*—One hundred and fifty students attended the recent session at this institution. From the annual circular, we learn that the faculty have lately established a Chair of *Physical Diagnosis and Pathological Anatomy*, to which Professor A. Worcester has been appointed. The vacancy in the chair of Theory and Practice, caused by the resignation of Professor J. P. Kirtland, has been filled by Professor John Moorhead.

*Proceedings of the Medical Society of the State of Tennessee.*—The thirteenth annual meeting of this Society, was held at Nashville in the month of May, of the current year. The following officers were elected: Dr. A. H. Buchanan, of Columbia, President; Dr. Geo. Thompson, of Rutherford county, Vice President; Dr. J. Shelby, Treasurer; Dr. Waters, Corresponding Secretary; and Dr. R. Martin, Recording Secretary. The pamphlet which contains an account of the proceedings, is taken up mainly by an essay on the Theory and Pathology of Fever, by Dr. A. H. Buchanan. This is a learned and well-written paper, presenting, however, views on the subject of fever to which we cannot altogether subscribe.

*Ruschenberger's Series of Natural History—Reptiles and Fishes.*—The Fourth Book of this useful series has lately been issued. We are glad to learn that it has reached an extensive circulation.

*American Medical Library and Intelligencer.*—We regret to learn that this publication is suspended, and that we are, at least temporarily, to lose Professor Dunglison from the editorial corps. The Library has afforded the profession, at a cheap rate, a large amount of sterling matter, and the Intelligencer, we have great pleasure in saying, has been always conducted with courtesy and ability.

